# The Prevalence of Minimal Hepatic Encephalopathy in an Outpatient Hepatology Clinic

**DOI:** 10.5152/tjg.2023.21881

**Published:** 2023-05-01

**Authors:** Deniz Eyice, Ayşe Merve Ok, Abdullah Sonsuz, Mustafa Şükrü Şenocak, Meliha Nur Durak, Sebati Özdemir, Billur Canbakan, Murat Tuncer, Ibrahim Hatemi

**Affiliations:** 1 Department of Internal Medicine, Cerrahpaşa School of Medicine, İstanbul University-Cerrahpaşa, İstanbul, Turkey; 2 Division of Gastroenterology, Department of Internal Medicine, Cerrahpaşa School of Medicine, İstanbul University-Cerrahpaşa, İstanbul, Turkey; 3 Department of Biostatistics, Cerrahpaşa School of Medicine, İstanbul University-Cerrahpaşa, İstanbul, Turkey

**Keywords:** Cirrhosis, critical flicker frequency test, minimal hepatic encephalopathy, psychometric test

## Abstract

**Background:**

Minimal hepatic encephalopathy can only be detected by specific psychometric or neuropsychological tests. We aimed to determine the prevalence of minimal hepatic encephalopathy in a hepatology outpatient clinic of a tertiary center.

**Methods:**

A total of 82 patients with chronic liver disease were involved prospectively in this study. Control groups consisted of healthy volunteers (n = 123) and chronic renal failure patients (n = 28). We used 2 different methods to detect minimal hepatic encephalopathy. First method was a battery of 5 psychometric tests (number connection tests A and B, digit symbol test, serial dot test, line tracing test) which was filled by all patients. The second method was critical flicker frequency test. Both methods were used in the whole group (n = 233). We applied linear regression analysis to the results of psychometric tests of healthy volunteers to establish equations to calculate the expected values of each test. Test results of the patients were evaluated according to the expected results obtained from these equations.

**Results:**

The prevalence of minimal hepatic encephalopathy detected by psychometric tests and critical flicker frequency test was 13% and 14%, respectively. When the positivity of both tests was deemed necessary to diagnose minimal hepatic encephalopathy, the rate of minimal hepatic encephalopathy was 3.6% (n = 3) in a chronic liver disease patient group.

**Conclusion:**

Minimal hepatic encephalopathy is a difficult clinical condition to diagnose, and it is more appropriate to use psychometric tests and critical flicker frequency test together.

**Main Points**

- Minimal hepatic encephalopathy (MHE) is an important complication of cirrhosis that can alter quality of life of the patient. It is difficult to diagnose it because subtle clinical findings can be overlooked in daily practice. Minimal hepatic encephalopathy can be diagnosed only by psychometric and neurophysiological tests.
- Electrophysiological and psychometric tests can be evaluated together for a more accurate diagnosis of MHE.
- To scan patients with cirrhosis for MHE, psychometric hepatic encephalopathy score nomograms for each test should be produced from healthy population.

## Introduction

Hepatic encephalopathy (HE) is a neuropsychiatric syndrome caused by impairment of the function of central nervous system caused by liver failure and/or portosystemic shunts. Hepatic encephalopathy may cause subclinical psychiatric abnormalities; sleep disorders, alteration in consciousness and neurological disorders.^1,2^ Patients who have minimal hepatic encephalopathy (MHE) do not have signs of overt HE; nevertheless, symptoms and signs of cognitive dysfunction such as decreased attention, memory problems, or decreased learning capacity may occur.^3,4^ The frequency of MHE is reported in a wide range such as 20% to 80% in chronic liver disease (CLD) patients.^5^ Patients with MHE may have difficulties to perform tests that require psychomotor speed, visual perception, and attention. It is important to investigate MHE in people with CLD because it affects quality of life of the patient and may also cause traffic and/or work accidents.^4,6-8^

In this study, we aimed to evaluate the frequency of MHE in CLD patients and compare the psychometric and neurophysiologic test findings with chronic renal failure (CRF) and healthy control groups in a tertiary hepatology outpatient clinic.

## MATERIALS AND METHODS

The CLD patients who were followed by the hepatology outpatient clinic were prospectively enrolled in the study for a period of 8 months. Patients who have had the diagnosis of HE in the past, patients who had symptoms or signs of overt HE (OHE) (defined as to detect signs of encephalopathy which is higher than stage 1 encephalopathy according to the West-Haven criteria)^5^ at the time of this study, patients who had a history of upper gastrointestinal bleeding or spontaneous bacterial peritonitis in the last 2 weeks, and patients who were using lactulose or psychoactive drugs were not included in the study. In addition, patients who had neurological or psychiatric illness (such as Alzheimer’s disease, Parkinson’s disease, mania, schizophrenia, etc.), patients who had visual impairment, patients who had liver transplantation, shunt surgery, or transhepatic portosystemic shunt were also excluded. Other exclusion criteria were comorbid conditions (decompensated heart failure, renal insufficiency, chronic pulmonary disease, active malignancy under treatment), being illiterate, alcohol intake of more than 50 g per day during past 3 months, and the presence of a condition that can cause non-hepatic metabolic encephalopathy such as hyponatremia, hypocalcemia, hyperglycemia, and hypoglycemia.

We determined 2 control groups which were healthy control group and diseased control group. Healthy control group consisted of medical students and hospital staff. For diseased control group, we recruited patients with stage 3 or 4 CRF. The patients in CRF group were not undergoing hemodialysis. These groups consist of patients with glomerular filtration rate of 15-60 mL/min/1.73 m^2^.

For control groups, we used these exclusion criteria: the presence of CLD, neurological disease, psychiatric disease, or other disease which may affect cognitive function, alcohol intake >50 g per day over the past 3 months, to be illiterate.

Before testing for MHE, we performed mini mental test for every patient if the score was less than 24, we exclude the patient from the study. If the patient was eligible, we performed psychometric tests and flicker frequency test in the same day.

### Psychometric Tests

Five psychometric tests such as number connection test-A (NCT-A) and number connection test-B (NCT-B), digit symbol test (DST), serial dotting test (SDT), and line tracing test (LTT) were applied to all participants. These paper-pencil tests were developed and published by Weissenborn et al^4^ as psychometric encephalopathy tests (PHES) to use for the diagnosis of HE. We applied all tests in a calm and sufficiently enlightened room.

The results of DST are given as scores, whereas the results of NCT-A, NCT-B, and SDT test are given as seconds. The results of LTT are measured with time to complete the test (LTTt, seconds) and with an error score (LTTe). In all tests, except DST, the score is inversely proportional to success. In other words, as the score decreases, success increases. In DST test, the higher the score, the more successful the patient is.

### Interpretation of Psychometric Tests

Psychometric tests might be influenced by the education level of the subject. We tried to overcome this bias by trying to choose similar level of education between groups. In addition, we excluded subjects who have more than 15 years of education from diseased control and healthy control groups when we are calculating the equations for the expected values of each psychometric test because the highest level of education in CLD patient group was 15 years. In previous studies, test results with a score ≤-2SD were accepted as abnormal.^4,9^ According to the calculated expected values for psychometric tests, we accepted that subjects who get a Z score which is less than ≤ -2 SD in at least 2 of 5 tests had MHE.

### Critical Flicker Frequency Test

**Figure 1.**
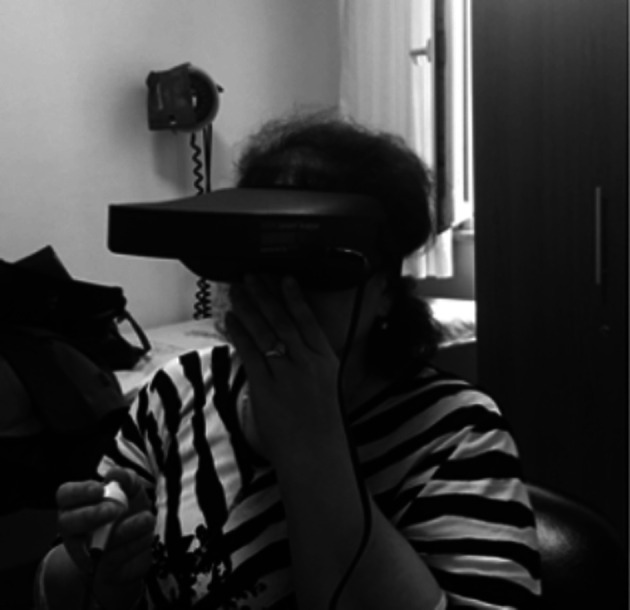
Critical Flicker Frequency test (permission obtained from the patient).

The critical flicker frequency (CFF) test is a neurophysiological test method based on the evaluation of the critical vibration frequency and intermittent stimuli presented to the eye are perceived as separate only if the presentation rate is below a certain threshold.^10^ The Hepatonorm Analyzer (nevoLAB GmbH, Germany), developed for the application of this test, is a noninvasive method, consisting of a Goggle-like applicator and a button holder in the hand of the patient. During the test, a virtual diode light emanating from a light source 12 m away is simulated and intrafoveal stimulation is applied to the patient. Beginning with 60 Hz, the frequency of light is reduced, and the test is performed. The patient is being asked to press the button when he saw the light flickering in the Google-like applicator (Figure 1). The process and the test were explained to the participants in detail and then the test was carried out after the staff was confident that the subject understood what he/she should do. The average value of 7-9 measurements made was recorded.

### Criteria Used in the Diagnosis of Minimal Hepatic Encephalopathy

Minimal hepatic encephalopathy was considered in patients if the mean value of the patients in the CFF test was <39 Hz.^11^ For psychometric tests, patients who have ≤2 SD in at least 2 of 5 tests were considered to have MHE.

### Patient Evaluation

Main clinical and demographic information were collected from the patients and medical records. Then, physical examination was performed, biochemical parameters (creatinine, aspartate aminotransferase [AST], alanine aminotransferease [ALT], total bilirubin, direct bilirubin, albumin, sodium, prothrombin time [PT], International Normalized Ratio [INR]) and serologic markers (HBsAg, anti-HBs and anti-HCV) were noted. Abdominal ultrasonography and upper gastrointestinal endoscopy findings were recorded from the medical charts and clinical visit notes. In addition, complications related to the cause of liver cirrhosis or portal hypertension disease were recorded. The Child–Pugh score was used to determine the liver cirrhosis stage of the patients.^12^

The institutional review board and the Ethics Committee of İstanbul University Cerrahpaşa School of Medicine approved the study (02/140398). Written consent was obtained from all patients and volunteers.

### Statistical Analysis

Statistical analysis was performed using the statistical software package Statistical Package for the Social Sciences version 18.0 (SPSS Inc.; Chicago, IL, USA). Chi square, Kruskal–Wallis, and Mann–Whitney U tests were used to compare continuous and categorical data, and the results were tested at 95% confidence level. Spearman’s correlation analysis was used to evaluate the relationship between the 2 variables. The statistical significance between age, gender, and years of education was assessed by calculating Spearman’s correlation coefficient at 95% CI. Regression analysis was performed with variables that affect psychometric tests statistically. Psychometric tests were standardized with the results obtained from the regression equations for each test.

## Results

For the liver cirrhosis group, 167 patients were evaluated, and 82 patients were included in the study after elimination according to the exclusion criteria. A total of 72 patients (87.8%) were classified as Child A and 10 (12.2%) were classified as Child B.

One patient could not be tested in this group because he did not understand how to perform the CFF test. For the healthy control group, 123 participants were included, 4 participants could not make the LTT test, and 1 could not make SDT test. A total of 98 participants performed the CFF test. In the renal failure group, 28 patients were included, 1 participant could not complete the paper-pencil tests, and 5 participants could not make CFF test. When the CFF test was first started to perform in our clinic, we used the mean of 7 measurements for the first 9 healthy control as the software that we were using allowed 7 measurements to calculate the mean value, afterward 9 measurements were made for patients and controls.

The results of the psychometric tests of the healthy control group were analyzed by linear regression analysis. We observed that age and duration of education significantly affected test results. By using these parameters, we created an equation that will be used to calculate the expected value of the test result. We used the same method for each test. Test results of the patient group were evaluated according to the expected values obtained from these equations. The equations are shown in the Table 1.

In the healthy control group, mean duration of education year was higher in the young subjects (18-30 years) than in the rest of the group. In addition, as shown in Tables 1 and 2, we observed that younger subjects and subjects who had a higher educational level had positively affected test results except for LTT-e test. Paper-pencil test results were not affected by gender. Age was a significant factor for all paper-pencil tests, and the duration of education years was a significant factor for all paper-pencil tests except LTT-e. In Table 2, the correlations of psychometric tests between age and education years with R values were shown.

In Table 3, we showed general characteristics and test results of the CLD patients compared with the control groups. In Supplementary Table 1, findings of CLD group in 2 separate groups were summarized.

According to our criteria, 13% (11/82) of the patients were found to have MHE according to the results of psychometric tests. This rate was 7% (2/27) in the diseased control group. Among 5 tests, NCT-A was the most frequent abnormal test. Abnormal test results for the CLD, CRF, and healthy groups are given in Tables 4 and 5.

In our study, CFF positivity was present in 14% (12/81) of the CLD group. This rate in the healthy control group was 7.3% (7/95). The CFF positivity was found in 8% (2/23) in the diseased control group. In the CLD group, 50% (6/12) of the CFF-positive patients were completely successful in the paper-pencil test.

If we combine the results of 2 methods, MHE frequency in the CLD group was 24% (20/82). The frequency of MHE detected by psychometric tests was 13% (11/82), and the frequency of MHE detected by CFF was 14% (12/81). In total, 3% of 82 patients (3/82) were found to have MHE by both methods.

In CLD patients, there was no difference in terms of gender, education time, and etiology of liver disease according to the presence of MHE. The age was significantly higher in the group with MHE (*P* = .03). Nine of those positive for the paper-pencil tests were Child A, 2 of them were Child B. Eleven of those who were positive for CFF were Child A and 1 was Child B.

There was no significant difference between patients who have MHE in CRF patient group and CLD patient group according to the age (*P* = .163), gender (*P* = .590), or duration of education (*P* = .924).

## Discussion

In this study which was carried out in a state university hospital gastroenterology and hepatology outpatient clinic, we determined the PHES nomograms Turkish healthy group and the result of screening for MHE in CLD patients compared with healthy controls and CRF patients.

Minimal hepatic encephalopathy was first described in patients with portosystemic shunts as a condition of subclinical hepatic encephalopathy in the early 1970s. The hypothesis that mental changes occur before the initiation of neurological symptoms of HE has been proposed by Zeegen et al.^13^ Rehnstrom et al^14^ found significant intellectual impairments in a similar group of patients. Minimal hepatic encephalopathy is important for society because even a subclinical encephalopathy level is thought to affect driving capability by affecting attention and visual perception ability. Because of that, screening and treatment of MHE may reduce traffic accidents. Minimal hepatic encephalopathy diagnosis and treatment is also important because its treatment can improve mental function and prevent the progression to overt HE.

There is no gold standard test or method to diagnose MHE. To make the diagnosis of MHE neurophysiologic tests, computer tests or at least 2 of the psychometric tests should be positive.^15^ Critical flicker frequency test is also considered to be a sensitive method for the diagnosis of MHE.^16^ The PHES is usually used in daily practice.^17^ This battery consisted of 5 tests (NCT-A, NCT-B, DST, SDT, LTT).^4^ These tests should be standardized in a broad control group because arithmetic, literacy, and language skill differences can change test results.^17^ Test results are also influenced by age, gender, years of education, alcohol use, and vision skills of the subject.^18^ Validation studies have been carried out in Germany,^4^ Italy,^9^ Spain,^19^ Korea,^20^ China^21^, and India^22^ for standardization of psychometric encephalopathy. Although the results can be corrected according to age and education, it is more difficult to control the effect of other variables. Another problem is the decreasing sensitivity of these tests by frequent repetitions.^5^ There is no validation study for PHES in our country because of that we formed correcting equations by using test results of healthy volunteers and we evaluated test results of our patients by using these equations. In our evaluation, psychometric tests are adjusted according to age and education. The duration of the paper-pencil test is about 20 minutes, but they can show significant variations between patients. Although the tests are easy to apply, their interpretation is difficult. Our study showed that each psychometric test positivity rate ranged from 0% to 18.2%.

Age and educational levels are different between the groups. We adjusted the healthy control groups to overcome this problem. We excluded the subjects who have more than 15 years of education from healthy groups before the linear regression analysis because the upper limit of duration of education was 15 years in the patient group. After that, we created the equations using age and education level. Thus, normal values of each test were obtained from healthy group which were corrected according to the age and education level. Then, the tests of the patient group (patient who has liver disease) and the diseased control group (patient who has CRF) were compared with the normal values calculated for the same age and education level.

The application of the CFF test is simple and less influenced by variables such as education and age. In the previous study published by Kircheis et al^10^, 3% out of the healthy group (n = 261) of CFF assessments were found to be below normal. There are 2 values defined for CFF: 39 Hz^11^ and 38 Hz.^23^ It is known that in one of the premise studies from the German population, the CFF test distinguishes between HE and MHE when the threshold value is considered to be 39 Hz.^11^ In previous studies, it was reported that using the threshold of 39 Hz increased the sensitivity up to 97%, while specificity was about 94%.^10^ There are also other studies from the different European populations that used the cut-off level 39 Hz.^24,25^ In a recent study on the diagnosis of MHE reported from our country, the cut-off level of 39 Hz was used.^26^

The rate of MHE detected by paper-pencil tests was 13% in our study. The CFF test positivity was 14%. Minimal hepatic encephalopathy was diagnosed in 3 of 20 patients (15%) with both methods. If we accepted that the positivity of one psychometric test was enough for the diagnosis of MHE, the rate of MHE would be 40%. We found that a significant proportion of patients with liver cirrhosis without OHE were diagnosed as having MHE (28%). In our study, 71 patients were categorized as Child A (86%) and 10 patients as Child B (12%). Consistent with previous studies, the prevalence of MHE was significantly associated with liver function. Romero-Gómez et al^19^ reported the MHE rate 12 of 57 patients (21%) were in Child–Pugh class A and 23 out of 57 (40.3%) patients were in class B or C. Seo et al^20^ reported MHE rate 41 of the 160 patients with Child–Pugh grade A (20.2%); 26 of the 129 patients with Child–Pugh grade B (42.9%); and 6 of the 10 patients with Child–Pugh grade C (60.0%). Li et al^21^ reported the similar proportion of patients with MHE increased with the increase in the Child–Pugh grade.

A recent study from Turkey reported the frequency of MHE in 124 compensated cirrhotic patients (80% were Child–Pugh class A) was 30% with paper-pencil tests and 27% with CFF test.^26^ The prevalence is 16% when both methods were combined. There are 2 main biases in this study which are older age of cirrhotic patients and being less educated. The frequency of MHE in this study is about 2 times higher compared to our study. We used the norms of this study to interpret our paper-pencil test results; we detected that the rate of MHE was 12% (10/82), which was nearly the same as our result which was 13% (11/82). We cannot explain exactly the reason for this discrepancy. The degree of liver insufficiency cannot be a major factor since in both groups, child A patients were about 80%. We thought that the difference in education level can be a factor because the mean years of education is 8 years in our group, although more than 50% of the above-mentioned study had primary school education.

Because of the difficulties in interpreting psychometric tests, it is preferable to use electrophysiological tests that seem more appropriate for the diagnosis of MHE. Despite the presence of publications suggesting that the CFF test is a more useful test to diagnose MHE because it does not constitute a problem of standardization and implementation, the concordance between the results of the 2 methods in our study was low.

In our study, patients with CRF were also tested with both methods. These patients had stage 3 or 4 CRF but they were not undergoing hemodialysis. Because no serious metabolic changes like electrolyte imbalance are expected in this group of patients, we speculated that if there is any central nervous system (CNS) effect, it can be comparable to patients with CLD. We found that CRF patients had also minimal encephalopathy although the rate was lower than CLD. Best to our knowledge, the rate of MHE in CRF is not reported until now. This can be related to cognitive impairment caused by uremia, accumulation of toxic substances, or anemia may merit further investigation.

The major limitation of our study was the lack of standards for the interpretation of paper-pencil tests in our country when we finished the study, because of that test results were interpreted according to the statistical methods which were described in the Methods section. Another limitation is the difference in age and education levels between the patient and control groups. In the patient group, the highest education period was 15 years because of that we excluded 13 persons who have ≥15 years of education from the control group which decreased the number of healthy controls.

As a result, we believe that ideally electrophysiological and psychometric tests can be evaluated together for a more reliable diagnosis of MHE. In general, the diagnosis of MHE is complex and time-consuming. We think that screening for MHE in a busy outpatient clinic in daily practice is very difficult and is unsustainable. Future studies need to be carried out in a large patient group to clarify MHE’s definite diagnostic criteria and multicenter national studies are needed to validate widely applicable psychometric and neurophysiological tests.

## Figures and Tables

**Table 1.** Equations to Calculate the Expected Values of Each Psychometric Test

Tests	Equations
DST	52 039 - (0.647 × age) + (2.817 × education year)
NCT-A	27 641 + (0.481 × age) - (1.271 × education year)
NCT-B	63 465 + (0.826 × age) - (3.507 × education year)
SDT	42 400 + (0.400 × age) - (1.165 × education year)
LTT-t	24 041 + (0.649 × age) - (0.222 × education year)
LTT-e	1508 - (0.012 × age) - (0.052 × education year)

DST, digit symbol test; LTTe, error score of LTT; LTTt, time for completing line tracing test; NCT-A, number connection test-A; NCT-B, number connection test-B; SDT, serial dotting test.

**Table 2.** Correlations Between Each Psychometric Tests and Age and Duration of Education Year in the Healthy Control Group with R Values

	NCT-A	NCT-B	DST	SDT	LTT-t	LTT-e
Age	0.551**	0.521**	-0.510**	0.467**	0.506**	0.093
Duration of education	-0.490**	-0.589**	0.704**	-0.475**	-0.292**	-0.170*

*Spearman’s rho correlation is significant at the .05 level (2-tailed).

**Spearman’s rho correlation is significant at the .01 level (2-tailed).

DST, digit symbol test; LTTe, error score of LTT; LTTt, time for completing line tracing test; NCT-A, number connection test-A; NCT-B, number connection test-B; SDT, serial dotting test.

**Table 3.** General Characteristics of the Study Group and the Mean Value of the Psychometric Tests

Features	Healthy Controls	CLD	CRF	1-2-3	1-2	2-3	1-3
	(1)	(2)	(3)				
	Median (min max)	Median (min max)	Median (min max)				
Female (n (%))	66 (53)	35 (42)	13 (46)	ns			
Duration of education (year)	8 (1-15)	8 (5-15)	5 (1-15)	ns			
Age (years)	48 (18-70)	53.5 (18-70)	60 (26-70)	<.001	.004	.005	.001
NCT-A (s)	37 (15-86)	46 (17-165)	42.5 (0-120)	*P* = .003	.001	ns	ns
NCT-B (s)	64 (25-180)	84.5 (27-232)	82.5 (0-230)	<.001	<.001	ns	.004
DST (score)	44 (10-96)	32.5 (10-90)	30 (0-72)	<.001	<.001	ns	.001
SDT (s)	48 (27-141)	58 (30-170)	51 (0-141)	<.001	<.001	.019	ns
LTT-t (s)	47 (0-165)	56.5 (26-120)	51 (0-137)	*P* = .006	.002	ns	ns
LTT-e (count)	1 (0-9)	1 (0-8)	2 (0-12)	ns			

CLD, chronic liver disease; CRF, chronic renal failure, DST, digit symbol test; LTTe, error score of LTT; LTTt, time for completing line tracing test; NCT-A, number connection test-A; NCT-B, number connection test-B; SDT, serial dotting test.

0: healthy control, 1: liver disease, 2: renal disease.

0-1-2: Kruskal–Wallis test; 0-1, 1-2, 0-2: Mann–Whitney U tests.

**Table 4.** Psychometric Test Positivity in Patient Groups

Tests	CLD (n = 82)	CRF (n = 28)	Healthy (n = 123)	1-2-3	1-2^#^	1-3^#^	2-3^#^
	(1)	(2)	(3)				
NCT-A	15 (18.2%)	2 (7%)	3 (2.4%)	*P* < .001	ns	*P* < .001	ns
NCT-B	14 (17%)	4 (14%)	2 (1.6%)	*P* < .001	ns	*P* < .001	.01
SDT	12 (14.6%)	1 (3%)	5 (4.1%)	*P* = .016	ns	.010	ns
LTT-t	6 (7%)	1 (3%)	3 (2.4%)	ns			
LTT-e	3 (3%)	4 (14%)	10 (8.1%)	ns			

LTTe, error score of LTT; LTTt, time for completing line tracing test; NCT-A, number connection test-A; NCT-B, number connection test-B; SDT, serial dotting test, CLD, chronic liver disease, CRF, chronic liver failure.

^#^Chi square test.

**Table 5.** Positive Test Results of Patients in the Patient and Control Groups with Psychometric and CFF Tests

Results	CLD	CRF	Healthy	1-2-3	1-2^#^	1-3^#^	2-3^#^
	(1)	(2)	(3)				
1 test positive	22/82 (26%)	2/27 (7%)	18/123 (14.6%)	.023	.034	.031	ns
2 tests positive	5/82 (6%)	0	2/123 (1.6%)	ns			
3 tests positive	6/82 (7%)	1/27 (3%)	0	.011	ns	.004	ns
4 tests positive	0	1/27 (3%)	0	ns			
CFF test positive	12/81 (14%)	2/23 (8%)	7/95 (7.3%)	ns			

CLD, chronic liver disease; CRF, chronic liver failure; CFF, critical flicker frequency test.

^#^Chi-square test.

**Supplementary Table 1.** Psychometric Tests of the Patient Group

Child	Child A (A)	Child B (B)	A-B	Healthy Control-A	CRF-A
	72/82 % 87.8	10/82 % 12.2			
	Median (min-max)	Median (min-max)			
NCT-A (s)	44.5 (17-165)	48 (25-105)	ns^#^	.001^#^	ns^#^
NCT-B (s)	83.5 (27-195)	96 (44-232)	ns^#^	<.001^#^	ns^#^
SDT (s)	58 (30-170)	58 (42-90)	ns^#^	<.001^#^	.02^#^
DST (score)	32.5 (10-90)	31 (15-61)	ns^#^	<.001^#^	ns^#^
LTT-t (s)	53 (26-110)	74.5 (36-120)	ns^#^	.01^#^	ns^#^
LTT-e (count)	1 (0-8)	1.3 (0-4)	ns^#^	ns^#^	ns^#^
NCT-A positive	12	3	ns^	.001^	ns^
NCT-B positive	10	4	ns^	.001^	ns^
SDT positive	11	1	ns^	.012^	ns^
LTT-t positive	4	2	ns^	ns^	ns^
LTT-e positive	3	0	ns^	ns^	ns^
1 test positive	19	3	ns^	.043^	ns^
2 tests positive	4	1	ns^	ns^	ns^
3 tests positive	5	1	ns^	.006^	ns^
4 tests positive	0	0	ns^	ns^	ns^
CFF test positive	11/71	1/10	ns^	ns^	ns^

CRF, chronic renal failure, DST, digit symbol test; LTTe, error score of LTT; LTTt, time for completing line tracing test; NCT-A, number connection test-A; NCT-B, number connection test-B; SDT, serial dotting test, CFF, critical flicker frequency test, ns, non-significance.

^Chi-square test, ^#^Mann–Whitney *U* tests.
